# Label-free multimodal nonlinear optical imaging of needle biopsy cores for intraoperative cancer diagnosis

**DOI:** 10.1117/1.JBO.27.5.056504

**Published:** 2022-05-28

**Authors:** Lingxiao Yang, Jaena Park, Eric J. Chaney, Janet E. Sorrells, Marina Marjanovic, Heidi Phillips, Darold R. Spillman, Stephen A. Boppart

**Affiliations:** a University of Illinois at Urbana-Champaign, Department of Electrical and Computer Engineering, Urbana, Illinois, United States; b University of Illinois at Urbana-Champaign, Beckman Institute for Advanced Science and Technology, Urbana, Illinois, United States; c University of Illinois at Urbana-Champaign, Department of Bioengineering, Urbana, Illinois, United States; d University of Illinois at Urbana-Champaign, Carle Illinois College of Medicine, Champaign, Illinois, United States; e University of Illinois at Urbana-Champaign, College of Veterinary Medicine, Urbana, Illinois, United States; f University of Illinois at Urbana-Champaign, Cancer Center at Illinois, Urbana, Illinois, United States

**Keywords:** nonlinear optical microscopy, label-free imaging, intraoperative imaging, cancer diagnosis

## Abstract

**Significance:**

Needle biopsy (NB) procedures are important for the initial diagnosis of many types of cancer. However, the possibility of NB specimens being unable to provide diagnostic information, (i.e., non-diagnostic sampling) and the time-consuming histological evaluation process can cause delays in diagnoses that affect patient care.

**Aim:**

We aim to demonstrate the advantages of this label-free multimodal nonlinear optical imaging (NLOI) technique as a non-destructive point-of-procedure evaluation method for NB tissue cores, for the visualization and characterization of the tissue microenvironment.

**Approach:**

A portable, label-free, multimodal NLOI system combined second-harmonic generation (SHG) and third-harmonic generation and two- and three-photon autofluorescence (2PF, 3PF) microscopy. It was used for intraoperative imaging of fresh NB tissue cores acquired during canine cancer surgeries, which involved liver, lung, and mammary tumors as well as soft-tissue sarcoma; in total, eight canine patients were recruited. An added tissue culture chamber enabled the use of this NLOI system for longitudinal imaging of fresh NB tissue cores taken from an induced rat mammary tumor and healthy mouse livers.

**Results:**

The intraoperative NLOI system was used to assess fresh canine NB specimens during veterinary cancer surgeries. Histology-like morphological features were visualized by the combination of four NLOI modalities at the point-of-procedure. The NLOI results provided quantitative information on the tissue microenvironment such as the collagen fiber orientation using Fourier-domain SHG analysis and metabolic profiling by optical redox ratio (ORR) defined by 2PF/(2PF + 3PF). The analyses showed that the canine mammary tumor had more randomly oriented collagen fibers compared to the tumor margin, and hepatocarcinoma had a wider distribution of ORR with a lower mean value compared to the liver fibrosis and the normal-appearing liver. Moreover, the loss of metabolic information during tissue degradation of fresh murine NB specimens was shown by overall intensity decreases in all channels and an increase of mean ORR from 0.94 (standard deviation 0.099) to 0.97 (standard deviation 0.077) during 1-h longitudinal imaging of a rat mammary tumor NB specimen. The tissue response to staurosporine (STS), an apoptotic inducer, from fresh murine liver NB specimens was also observed. The mean ORR decreased from 0.86 to 0.74 in the first 40 min and then increased to 0.8 during the rest of the hour of imaging, compared to the imaging results without the addition of STS, which showed a continuous increase of ORR from 0.72 to 0.75.

**Conclusions:**

A label-free, multimodal NLOI platform reveals microstructural and metabolic information of the fresh NB cores during intraoperative cancer imaging. This system has been demonstrated on animal models to show its potential to provide a more comprehensive histological assessment and a better understanding of the unperturbed tumor microenvironment. Considering tissue degradation, or loss of viability upon fixation, this intraoperative NLOI system has the advantage of immediate assessment of freshly excised tissue specimens at the point of procedure.

## 1 Introduction

Cancer is the second leading cause of death in the United States^1^ and developing new technologies and methods for cancer diagnosis has been a major research field in biomedicine. For the diagnosis of cancer, especially in liver, lung, breast, prostate, and soft tissue tumors,^2^ it is a standard clinical practice to acquire and evaluate needle biopsy (NB) specimens before deciding on the treatment plan, such as the need for chemotherapy, radiation therapy, and/or surgical procedures.^2^ NB procedures include fine-needle aspiration, which often uses syringes to collect fluids for cytological evaluation, and core-needle biopsy, which collects tissue cores from suspicious regions.^2^^–^^5^ This study will focus on the collection and evaluation of NB core specimens, but the technologies and methods presented here could similarly be used to assess the cytologic content from fine-needle aspirations. NB core specimens are commonly fixed right after the acquisition and go through standard histological procedures including sectioning and staining.^2^ Although histopathology is the gold standard for cancer diagnosis, this conventional evaluation method often takes a day or more to generate the results.^6^^,^^7^ The time-consuming histological evaluation has become more of a concerning factor when the NB sampling is inadequate or non-diagnostic, which means the histology results of NB specimens fail to provide any diagnostic information, such as the presence of cancer cells. In this case, additional specimens are required, which further delays the diagnostic decisions.^2^^,^^8^ Therefore, there is a critical need to have a rapid screening and evaluation method of NB cores right after extraction and at the point-of-procedure.

In recent years, various optical imaging modalities have been demonstrated as potential tools to assess NB tissue cores, including confocal fluorescence microscopy,^8^ fluorescence structured illumination microscopy,^9^ optical coherence tomography (OCT),^10^^,^^11^ light-sheet microscopy,^12^ second-harmonic generation (SHG), and third-harmonic generation (THG) microscopy,^13^^–^^15^ and infrared (IR) microscopy and spectroscopy.^14^^,^^16^ Many studies involve the addition of chemicals such as exogenous fluorescent dyes,^8^^,^^9^^,^^12^^,^^15^ optical clearing agents,^12^^,^^13^ and fixation agents,^12^^,^^16^ which can be destructive to the tissue specimens and alter the tissue integrity, particularly if the optical imaging and sensing methods seek to measure metabolic, molecular, or dynamic changes that are occurring within the biopsied tissue. On the other hand, OCT was demonstrated as a label-free imaging tool for guiding the acquisition of NB cores,^10^ but the resolution of OCT limited its performance to visualizing only the larger microstructures in the tissue, which may further limit its capability in providing diagnostic information.^10^^,^^11^

For visualization of the unperturbed tissue microenvironment, label-free nonlinear optical imaging (NLOI) has been a rapidly developing approach in biomedical research with an expanding range of applications.^17^^,^^18^ For example, the nonlinear Raman microscopy and spectroscopy modalities, including coherent anti-Stokes Raman scattering^19^ and stimulated Raman scattering,^20^^,^^21^ can provide quantitative biochemical contrast. Multiharmonic generation microscopy such as SHG^17^^,^^22^^–^^24^ and THG^25^ highlight the non-centrosymmetric structures and the optical heterogeneities in the tissue microenvironment, respectively. The development of multiphoton autofluorescence microscopy^26^^–^^29^ has enabled various discoveries and applications of endogenous fluorophores among which flavin adenine dinucleotide (FAD), reduced nicotinamide adenine dinucleotide, and reduced nicotinamide adenine dinucleotide phosphate (NAD(P)H) are of significant importance for their direct roles in cell metabolism.^18^^,^^30^^–^^34^ Two-photon fluorescence (2PF) of FAD and NAD(P)H has been used for non-invasive label-free metabolic imaging.^18^^,^^30^^–^^34^ The redox ratio in these studies, defined by FAD/(FAD + NAD(P)H),^18^ was used to characterize cell metabolism. The relatively higher concentration of NAD(P)H, which yields a lower redox ratio, usually indicates relatively more glycolysis than oxidative phosphorylation under normoxic conditions.^18^ Comparatively, an increase in redox ratio was observed in cells under acute hypoxia.^18^ By combining 2PF with fluorescence lifetime imaging microscopy (FLIM) technology, many studies have shown their advantages in revealing the metabolic states of living cells and tissue.^35^^–^^37^ High-speed two-photon FLIM has been shown to capture fast metabolic dynamics, including the responsiveness to an apoptotic inducer, such as staurosporine (STS).^36^^,^^37^ Although many metabolic imaging studies have used 2PF, there have been only a few demonstrations of three-photon fluorescence (3PF) of NAD(P)H and FAD in unstained tissue imaging,^28^^,^^29^ mainly limited by the intrinsically weaker 3PF signals and the requirements on the excitation laser source.

Our laboratory has previously demonstrated a benchtop simultaneous label-free autofluorescence and multiharmonic (SLAM) microscopy system combing SHG, THG, 2PF, and 3PF.^38^^–^^41^ SHG provides fine details of the collagen fibers in the extracellular matrix and THG highlights cell membranes and the boundaries of lipid droplets. At the same time, 2PF and 3PF are used to visualize the distribution of FAD and NAD(P)H, respectively. We have shown that this multimodal imaging technique can provide rich information on the tissue microenvironment,^38^^–^^41^ not only visualizing various microstructures but also revealing metabolic profiles, which cannot be achieved by standard histology.^41^^,^^42^ Therefore, the SLAM technique, with its high multi-dimensional and spatially co-registered image datasets, can be a potential tool for real-time assessment and diagnosis in clinical applications, especially with the addition of artificial intelligence (AI)-enabled tumor classifications.^43^^,^^44^ Coupled with its metabolic imaging capability, SLAM microscopy has potential to enhance our understanding of tissue, especially the tumor microenvironment.

In more recent work, an upgraded intraoperative multimodal NLOI system was reported and compared with an earlier design and a benchtop SLAM system.^45^^,^^46^ We also presented preliminary results of its performance in intraoperative imaging of fresh canine NB cores and structural correlations with corresponding histology images.^46^ Here we aimed to demonstrate the potential of the multimodal NLOI system on animal models, prior to a clinical study involving human subjects. In this paper, we will further show that this intraoperative, label-free, multimodal NLOI system can provide not only the histology-like structural information of canine NB tissue cores at the point-of-procedure, but also quantification of the microstructures and metabolic profiles of NB cores taken *in vivo* from different sites, including the tumor, tumor margin, and normal-appearing regions adjacent to the tumor. We further demonstrate that this intraoperative NLOI system has the potential for longitudinal monitoring of the changes in tissue integrity and metabolic activity over time, which could open new directions of research and applications for intraoperative label-free NLOI.

## 2 Methods and Materials

In this study, all the NLOI results were acquired using a custom-built portable imaging system. The intraoperative imaging was performed during canine cancer surgeries and a total number of eight canine patients were recruited. Due to the limited availability of surgical specimens, and the restrictions on the intraoperative imaging time, the longitudinal monitoring of tissue degradation was demonstrated through rodent specimens in the laboratory. We used the rat model for tumor induction as it was an established animal model with which we had previous experience. The mouse liver biopsy imaging was chosen to shorten the time used for regions-of-interest (ROI) selection since liver tissue is more homogeneous compared to tumor tissues and is metabolically active.

### 2.1 Intraoperative NLOI System

**Fig. 1:**
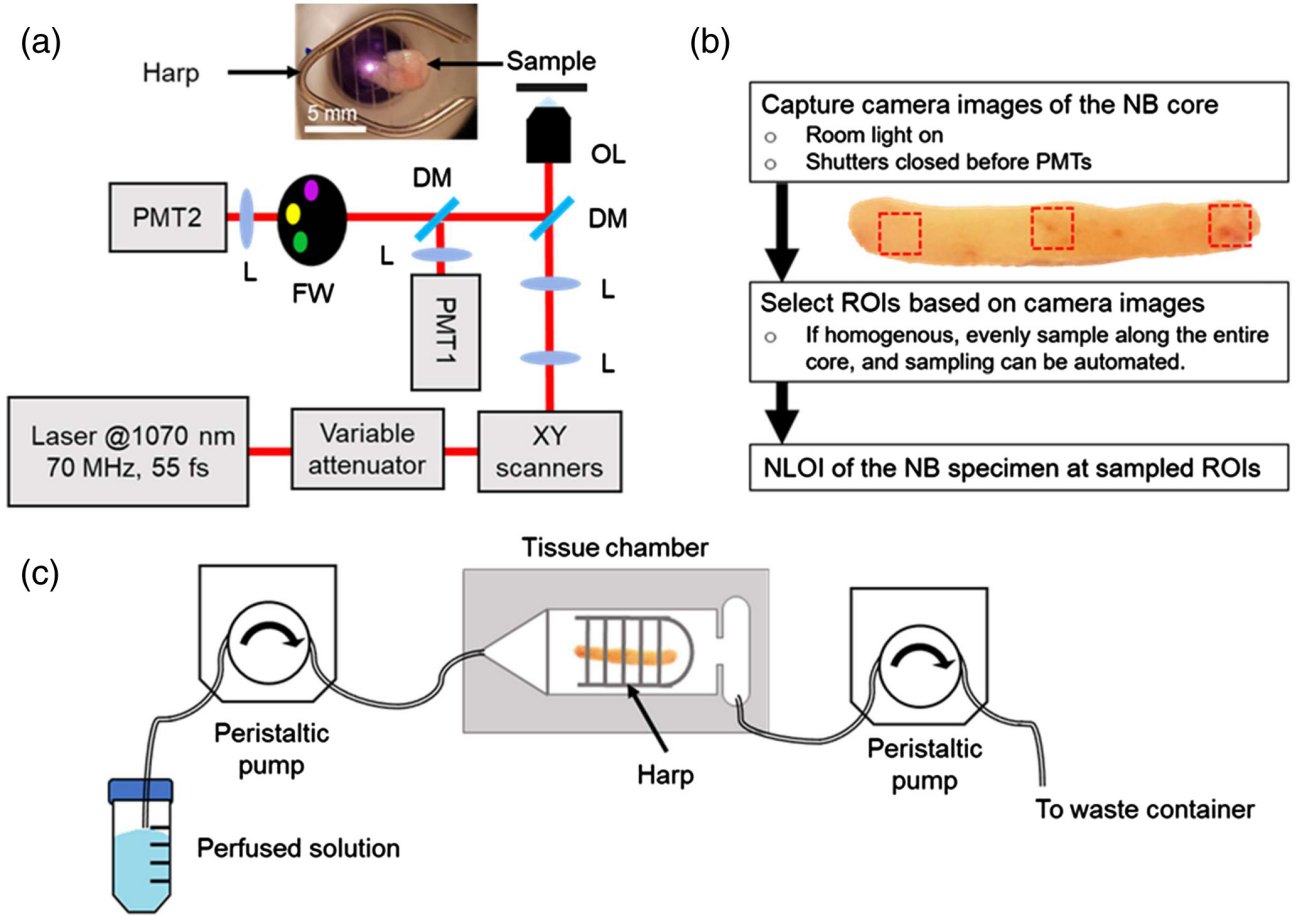
Experiment setup and workflow diagrams. (a) Optical setup of the intraoperative NLOI system. The laser delivered 55-fs pulses at 70 MHz at a center wavelength of 1070 nm. PMT1 was used to collect only the 3PF signals, and the THG, SHG, and 2PF signals were collected sequentially by PMT2 by switching a FW. The specimen was placed on a 2D stage and stabilized with a harp. The inset image shows a camera image of a canine mammary NB specimen acquired from the tumor margin and placed on a coverslip before being imaged. The image shows the laser beam position (bright spot) relative to the specimen, which corresponds to the first ROI. (b) Flowchart of the procedure for using the camera image of the NB specimen to guide the selection of ROIs to be acquired by the intraoperative NLOI system at the point-of-procedure. (c) Perfusion setup for longitudinal imaging of NB specimens. The tissue chamber with a coverslip bottom was placed on the stage of the same intraoperative NLOI system. PMT, photomultiplier tube; L, lens; FW, filter wheel; DM, dichroic mirror; OL, objective lens; ROI, region-of-interest.

The portable system used in this study for intraoperative imaging of fresh canine NB tissue cores was previously reported in detail.^46^ Figure 1(a) shows the schematic of the optical setup. For compactness and turn-key operation, a commercial fiber laser (Fidelity-2, Coherent, Inc.) was used to excite THG, 3PF, SHG, and 2PF channels simultaneously. The power at the sample plane was to be controlled at 20 mW before every imaging session using the variable attenuator. After the XY scanners, the beam was directed upward and focused on the specimen using a multiphoton objective lens of NA=1.05 and 25× magnification (XLPLN25XWMP2, Olympus, Inc.). The axial position of the objective lens was adjustable using a linear-piezo stage (SLC-24120, SmarAct GmbH), and the lateral position of the sample was controlled by a separate two-dimensional (2D) stage (M-545.2P, Physik Instrumente). The objective lens stage was used for fine-tuning the focus and estimation of the imaging depth by calculating the distance between stage positions at the image acquisition plane and the coverslip plane. The imaging depths were around 30 to 50  μm for the data presented in this paper. A metal slice anchor (harp, SHD-26H/15, Warner Instruments) on top of the specimen was used to secure the location of the specimen, as shown in the insert image in Fig. 1(a). The NLOI signals generated from the specimen were collected by two photomultiplier tubes (PMTs). We used a band-stop dichroic mirror (DM, ZT325/442rpc, Chroma Technology, Corp.) to reflect the 3PF channel signals from about 420 to 470 nm. A spectral filter (FF01-451/106, Semrock, Inc.) was placed in front of PMT1 [Fig. 1(a)] to further remove any residual wavelengths outside the 3PF channel spectral band. While PMT1 acquired 3PF signals, PMT2 collected signals from THG, SHG, and 2PF sequentially by switching a motorized filter wheel (FW). A four-channel image of 600×600  pixels was acquired at a 20 Hz line-rate within 1.5 min using the combination of sequential and simultaneous detection. The lateral and axial resolutions were characterized to be around 0.56 and 2.8  μm, by imaging fluorescent microspheres of 100-nm and 1-μm diameters under the 2PF channel, respectively.^46^ The maximum single field-of-view (FOV) of the imaging system was measured to be around $550\times 550\;{\mu m}^{2}$ (800×800  pixels) by imaging a reflective grid target (R1L3S3PR, Thorlabs, Inc.).^46^ The image area was variable by changing the scanning parameters, namely the pixel size and the pixel number, while the line rate was kept the same at 20 Hz. The FOV scanned in the intraoperative cancer imaging and rat mammary tumor NB core imaging was around $350\times 350\;{\mu m}^{2}$ (500×500  pixels) to capture as many features as possible within limited imaging time, while the FOV scanned in the longitudinal imaging of normal liver NB cores was around $210\times 210\;{\mu m}^{2}$ (600×600  pixels) since liver tissue is relatively more homogeneous, and decreased pixel-dwell time reduced the possibility of photodamage during continuous and extended imaging.

### 2.2 Canine NB Core Specimens

In this study, eight canine patients with owners’ consent were recruited from the Veterinary Teaching Hospital at the University of Illinois at Urbana-Champaign. There were four liver cancer cases, two soft-tissue sarcoma cases, and one each for lung and mammary tumors. The patient demographic information is listed in Table S1 in the Supplemental Material.

During canine cancer surgeries, NB tissue cores were taken using a 14-gauge manual core biopsy needle (Tru-cut, Merit Medical, Inc.), which resulted in NB cores of around 1.6 mm (diameter) × 12 mm (length) with some degrees of variation in size due to the stiffness of different tissue types and techniques of different surgeons.^46^ Before the surgical resection of the cancerous tissue mass, NB cores were taken *in vivo* by a veterinary surgeon from several tissue regions including the tumor, tumor margin, and the normal adjacent sites identified by the surgeon. These NB cores were then extracted and imaged by the intraoperative NLOI system at the point-of-procedure. After the surgical resection of the tumor mass, additional NB core specimens were acquired from the *ex vivo* tumor for imaging.

### 2.3 Intraoperative Imaging

The NB cores that were acquired *in vivo* were immediately imaged by the intraoperative multimodal NLOI system while the surgeon continued the surgical procedure to remove the tumor. Due to the time limit during surgical procedures, for each NB specimen, a 30-min imaging time was available, which allowed for the acquisition of around 10 to 15 FOVs. However, in some cases, there was only a 5- to 10-min interval between the *in vivo* extraction of NB specimens from different regions before the bulk surgical removal of the tumor, particularly for the tumor margin and the normal-appearing regions adjacent to the tumor. In these cases, not every *in vivo* acquired NB specimen was imaged immediately. The additional NB core specimens acquired from the *ex vivo* resected tumor were imaged immediately after the tumor extraction by the same system. After imaging, all NB specimens were placed in a formalin solution and underwent standard hematoxylin and eosin (H&E) histological processes. A flowchart of the detailed intraoperative imaging procedure is included in Fig. S1 in the Supplemental Material.

The inset image in Fig. 1(a) shows the top view of a canine mammary NB tissue core on a coverslip and stabilized by a harp. Due to the limited time of imaging during the surgery, the selection of ROIs was based on the top-view camera image taken before each imaging session. As illustrated in Fig. 1(b), the camera image can be readily captured by a smartphone, capturing the harp with fixed wire spacing of 1.5 mm, the NB specimen, and the laser position [bright spot in Fig. 1(a)]. The use of smartphones can be replaced by using cameras with high sensitivity at both visible and near-IR wavelength regimes for better reliability and reproducibility. The laser position was used to mark the beginning position of imaging and the harp spacing was used to estimate the distance between ROIs, which was used for the computer control of the motorized 2D stage. Usually, the NB specimen appeared to be homogenous in the camera image, in which the ROIs were equally spaced along with the specimen. However, if the camera image of the NB specimen showed different regional features (e.g., color, density, texture), the ROIs were selected to cover the visually distinguishable features.

### 2.4 Longitudinal Imaging

To study the longitudinal changes in the tissue microenvironment, a tissue chamber with perfusion capabilities was added to the intraoperative imaging system. The schematic for the perfusion system and tissue chamber is shown in Fig. 1(c). The tissue chamber (RC27, Warner Instruments) with a metal harp for stabilizing the position of the NB specimen was placed on top of the 2D stage in the intraoperative NLOI system. Two peristaltic pumps (Masterflex L/S) were used for perfusion, including solution input and waste output. The perfusion rate was controlled to be 1.5 to 2  mL/min. Healthy 4- to 5-month-old wild-type male mice (C57, Jackson Laboratory) were euthanized with ${\mathrm{CO}}_{2}$ asphyxiation. One mouse was used in each imaging experiment. The liver was surgically exposed and NB specimens were extracted using the same 14-gauge needle described in Sec. 2.2. The specimens were immediately placed in cold saline and transferred to the tissue chamber perfused with phosphate buffer solution (PBS). A concentration of 1 mM STS (Sigma-Aldrich) in dimethyl sulfoxide was diluted to 500 nM using PBS before the imaging experiment and kept on ice. The use of STS in this work followed the procedure reported in previous work which showed the induction of apoptosis in cultured cells.^36^^,^^37^

### 2.5 Rat Mammary Tumor Induction

Rat mammary adenocarcinoma cells (MAT B III, CRL-1666, ATCC), $1\times 10^{6}\text{ cells}/\mathrm{mL}$ in 100  μL McCoy’s media (Thermo Fisher), were injected subcutaneously into a healthy rat (Hsd: SD, Envigo) at the lower flank region, and after 2 weeks a tumor reached a size of approximately $5\times 5\times 10\;{\mathrm{mm}}^{3}$. The animal was euthanized with ${\mathrm{CO}}_{2}$ asphyxiation. The tumor was then surgically exposed and NB specimens were extracted using the 14-gauge needle as described in Sec. 2.2.

### 2.6 Image Acquisition, Postprocessing, and Statistics Analyses

The imaging system together with all the electronic and mechanical components was automated by a LabVIEW (National Instruments) program, which controlled the 2D stage, objective focusing stage, and FW. Details of the data acquisition were previously described.^46^ Grayscale images of individual channels were displayed during the acquisition. The four-channel images were merged, overlaid, and pseudo-colored using ImageJ (National Institutes of Health). The image display contrast was adjusted also using ImageJ by saturating the pixels with the top 5% of the highest intensities.

Quantitative analyses of the collagen fiber structures visualized by the intraoperative NLOI of NB tissue cores in Sec. 3.1.2 used a Fourier domain frequency filtering method^47^ on the SHG channel intensity images. Although there are well-known morphology-based processing methods for SHG images,^48^^,^^49^ these methods often involve iterative algorithms which can be time-consuming and largely rely on the image signal-to-noise ratio (SNR). The Fourier domain analysis method used in this work is noniterative and thus less computationally demanding. This method is also less dependent on the signal strength, meaning less subject to errors from intensity fluctuations and thresholding variations.^47^

MATLAB 2021b (MathWorks) was used for all the statistical analyses described in Secs. 3.1.3 and 3.2. The violin plots were generated using a modified code from an open-access code source.^50^ The interquartile range (IQR) was calculated as the difference between the 25th and 75th percentile bounds. The statistical significance was determined by the Kruskal–Wallis test and a p-value over 0.05 was considered to be not significant. The auto-segmentation method used in Sec. 3.2 was the *Cellpose* algorithm through its publicly available graphical user interface (GUI).^51^ The segmentation was performed on grayscale 2PF images with an input parameter of estimated cell size for each longitudinal imaging dataset, and the generated segmentation binary mask was applied to all four channels for quantitative analyses.

## 3 Results and Discussions

### 3.1 Intraoperative Visualization and Characterization of the Canine Tissue Microenvironment

#### 3.1.1 Histology-like label-free multimodal visualization

**Fig. 2:**
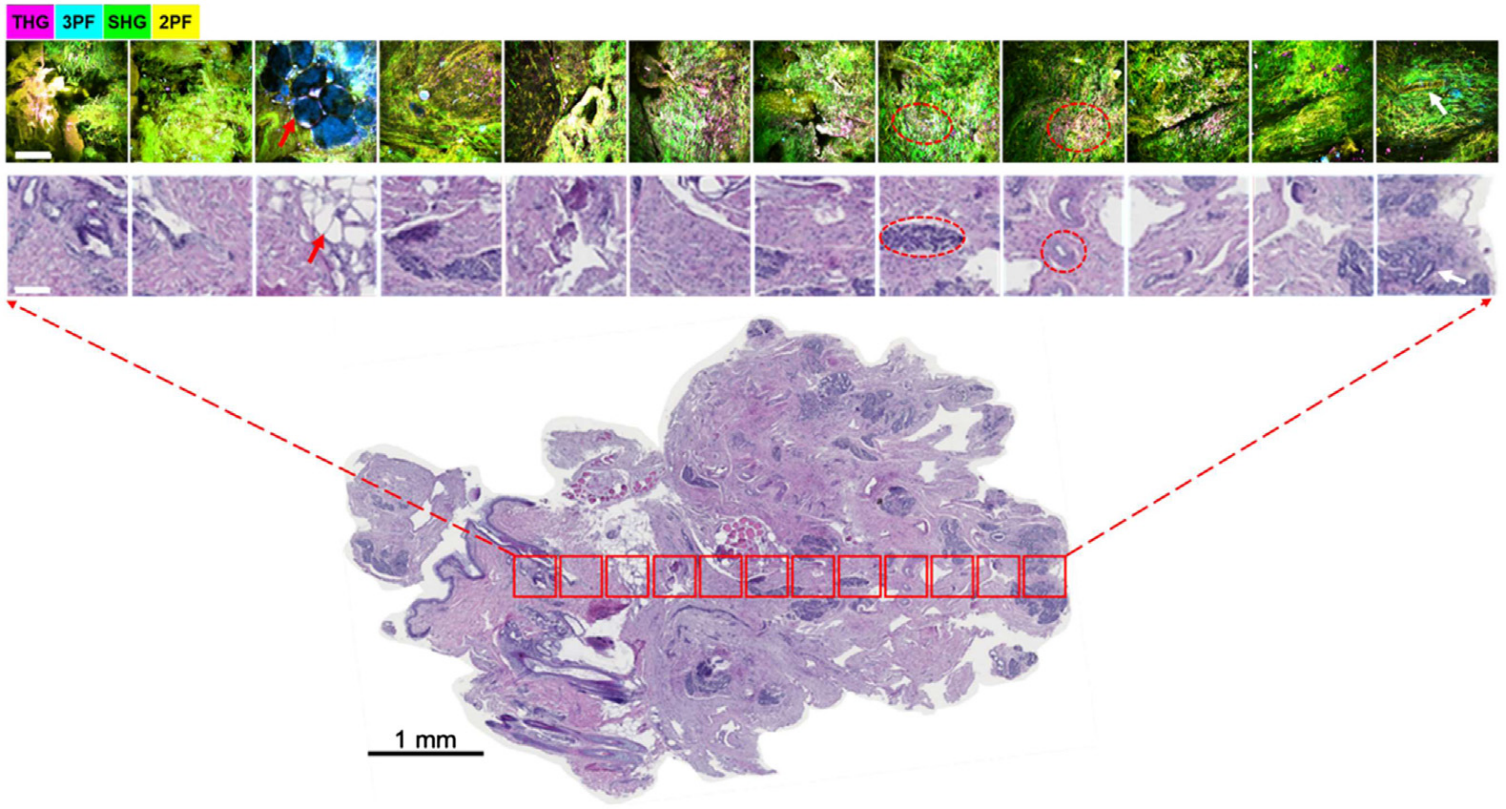
Intraoperative label-free multimodal NLOI of a fresh NB specimen acquired (biopsied) *in vivo* from a canine mammary tumor margin. Twelve ROIs (top) were acquired within 30 min and then correlated to the H&E histology image (bottom) of the entire NB specimen. Red arrows indicate an adipocyte cluster; red-dashed circles show examples of high-THG cells; white arrows mark a lobule. Scale bars in the top two rows of images represent 100  μm.

Using the ROI selection method described in Sec. 2.3, eight canine tumors, including liver, lung, and mammary tumors, and soft-tissue sarcoma were imaged. Figure 2 demonstrates the NLOI results and the correlated histological images at the ROIs selected based on the camera image representatively shown in the inset of Fig. 1(a). The specimen was taken *in vivo* from the margin region of a canine mammary tumor which was later diagnosed to be a grade-I multicentric mammary adenocarcinoma with solitary fibroadnexal hamartoma. The ROIs of $350\times 350\;{\mu m}^{2}$ were spaced 0.4 mm apart with the bright spot in Fig. 1(a) marking the start of the imaging, which corresponds to the left-most red box in Fig. 2 and the corresponding histology image. After imaging, each NB core was placed in the embedding cassette and secured using sponges before fixation. While every effort was taken to correlate the NLOI images of fresh specimens with the histology images of the fixed tissue, the orientations of the small tissue specimens were not perfectly preserved at the cellular scale, which may have affected the correlation accuracy. Although the distortions due to sample handling during the histology procedure were inevitable, structural correlations between the histology image and the NLOI results were still possible. For instance, the appearance of adipocytes (red arrows) and glandular lobules (white arrows) embedded in the dense fibrous stroma can be found in both NLOI and histology images. Scattered clusters of cells highlighted by the THG (magenta) channel (red dashed circles) in the NLOI results were later attributed to infiltrating epithelial cells, lymphocytes, and plasma cells in the tumor stroma as described in the veterinary pathological report. Figure S2 in the Supplemental Material gives other examples of intraoperative NLOI results correlated with histology using camera-image guidance, and an enlarged version of the NLOI results in Fig. 2 is shown in Fig. S3a in the Supplemental Material.

#### 3.1.2 Fast characterization of collagen structures

**Fig. 3:**
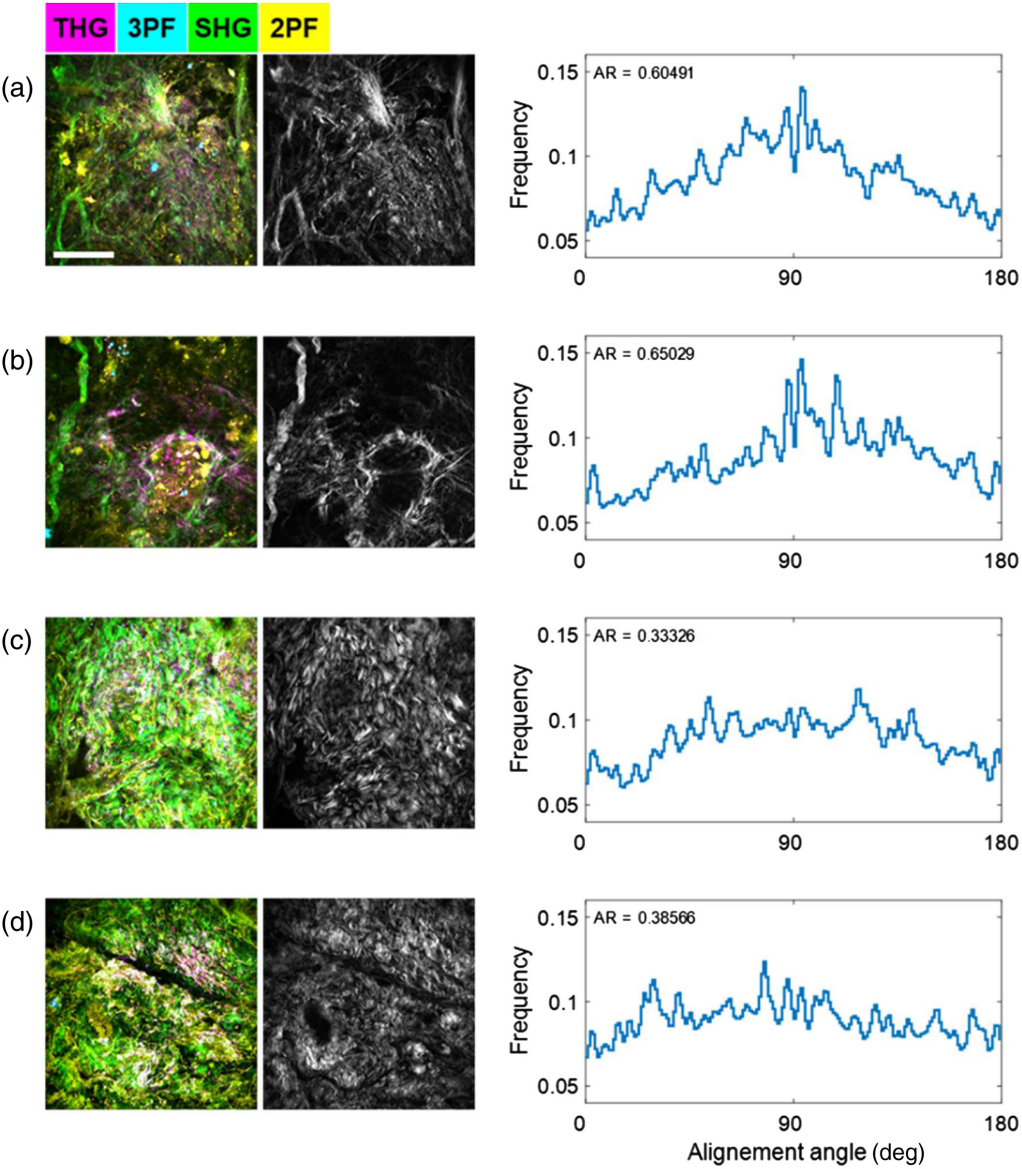
Collagen structural analysis by intraoperative label-free multimodal NLOI of canine mammary tumor (a, b) and margin (c, d) biopsies taken *in vivo*. Grayscale images in (a–d) are SHG images visualizing the collagen structures and a fast Fourier-domain analysis method^47^ was used to compute the distributions (right-most column) of the collagen fiber angles in the FOV and the AR. Scale bar represents 100  μm.

Figure 3 compares the Fourier analysis results of the canine mammary tumor (a, b) and tumor margin (c, d) SHG images (grayscale). The tumor margin images were from the same dataset shown in Fig. 2, and the mammary tumor images were from Fig. S3 in the Supplemental Material. The histograms in the right column of Figs. 3(a)–3(c) show the distribution of fiber angles obtained by rotating an angular Fourier filter in the frequency domain, and the fiber alignment ratio (AR) was calculated by dividing the peak value by the mean value in the histogram, which represents the extent of distinction of the peak, i.e., the dominant angle. As a result, the collagen fibers in the tumor SHG images [Figs. 3(a) and 3(b)] have more distinguishable angular distribution peaks with higher AR values compared to the analysis results for tumor margin [Figs. 3(c) and 3(d)]. This is consistent with the visual interpretation of the SHG intensity images, since the collagen fibers in the tumor SHG images have more distinct orientations, forming nest-like structures surrounding tumor cells, whereas the fibers in the tumor margin are more randomly orientated in a form of a dense network. Although the analyses were done in the post-processing procedure, they can also be incorporated in the image acquisition control program to provide the quantitative collagen structural analysis at the point-of-procedure.

#### 3.1.3 Metabolic profiling potentially at point-of-procedure

**Fig. 4:**
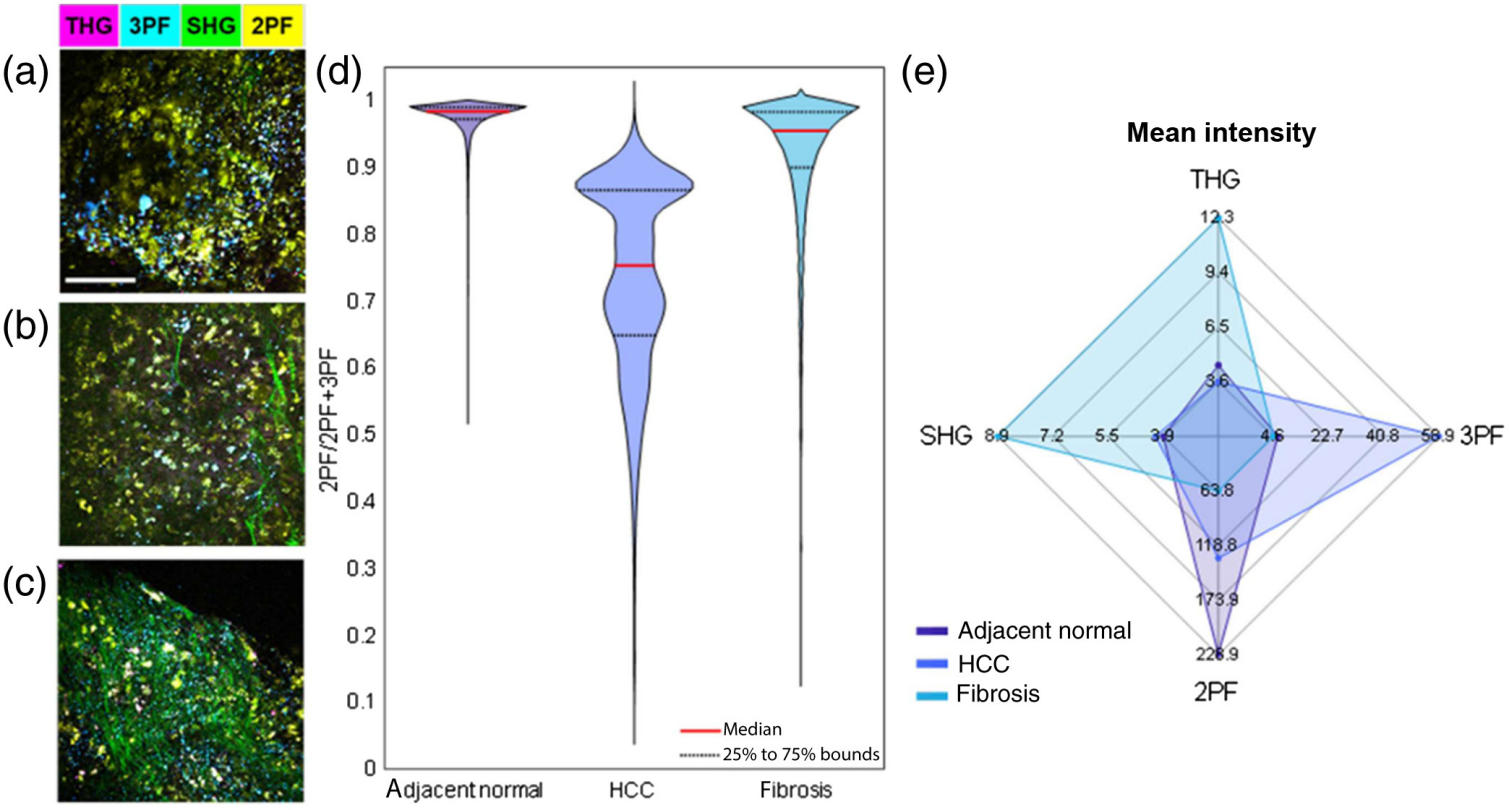
Label-free multimodal NLOI of *in vivo* biopsy taken from (a) normal-appearing area adjacent to HCC tumor, (b) HCC tumor, and (c) liver fibrosis. (d) ORR distributions from images (a–c) showing distinct metabolic profiles. The median values were marked with red solid lines and the gray dashed lines mark the 25th to 75th percentile bounds. The Kruskal–Wallis test was performed and the p-values among each group were below 0.001. (e) Radar plot of the liver biopsy NLOI results (a–c) using the averaged pixel intensity of each channel.

This intraoperative NLOI platform can also reveal metabolic information in the tissue microenvironment of the fresh NB specimens. The potential for metabolic profiling at the point-of-procedure was demonstrated through intraoperative NLOI of three different types of canine liver NB specimens. Figures 4(a)–4(c) show NLOI of canine NB specimens taken *in vivo* from a normal-appearing region adjacent to a hepatocellular carcinoma (HCC) tumor [Fig. 4(a)], the HCC tumor [Fig. 4(b)], and a region of liver fibrosis [Fig. 4(c)]. The metabolic profiles are represented by the ORR [ORR = 2PF/(2PF + 3PF)] distributions, which have been used to approximate the ratio of FAD/(FAD + NAD(P)H). As shown in Fig. 4(d), the HCC NB specimen had a lower average value and a wider variation range of ORRs compared to the values of the NB specimens from the normal adjacent region and a region of liver fibrosis. As introduced in Sec. 1, the lower ORR reflects a relatively higher concentration of NAD(P)H, which further indicates a preference for glycolysis over oxidative phosphorylation in cancer cell metabolism, known as the Warburg effect.^52^

The intensity contribution of each channel for each type of tissue was also determined. THG mainly highlights the membranes of cells and lipid droplets, but lipids usually produce stronger 3PF than that from the cells. In terms of fibrous structures, collagen fibers are highlighted by SHG, and elastin fibers appear in the 2PF channel, which mainly displays the FAD content in cells. Figure 4(e) shows a radar plot of the mean intensity of each NLOI channel in the adjacent normal, HCC, and fibrotic liver regions [Figs. 4(a)–4(c)]. Compared to the normal-appearing region adjacent liver, the HCC tumor NB specimen showed higher 3PF from the NAD(P)H autofluorescence in the tumor cells, since there was less lipid content observed in the multimodal image [Fig. 4(b)]. The dominance of 2PF signals in the normal adjacent liver NB specimens was attributed to the 2PF of FAD in hepatocytes. The collagen fiber content in fibrotic liver tissue can be found prominent in both the radar plot and the multimodal image [Fig. 4(c)]. The SHG of collagen in HCC was marginally stronger than that in the normal adjacent liver tissue, which can be attributed to the nested fiber structures shown in Fig. 4(b). Just as with the analysis method of collagen structures demonstrated in Sec. 3.1.2, metabolic profiling can also be integrated into the multimodal image acquisition software. Therefore, this intraoperative label-free multimodal NLOI system has the potential to perform metabolic profiling at the point-of-procedure.

### 3.2 Longitudinal Monitoring the Tissue Integrity

During the intraoperative NLOI of canine NB specimens, the time of imaging was largely limited by the surgical procedure. As described in Sec. 2.3, in some cases, not every NB specimen could be imaged immediately due to the acquisition time of the NLOI system. Though the NB specimens were all put in tissue culture media (specified in the table in Fig. S1 in the Supplemental Material) and temporarily stored on ice, the degradation of tissue integrity and these effects on the NLOI results remained uncertain. Therefore, the same intraoperative NLOI system was used to investigate the longitudinal changes in the optical signatures of the tissue microenvironment of fresh NB specimens, mainly regarding changes associated with metabolic parameters. To better sustain the NB specimens, a tissue chamber with a perfusion system was set up and used in conjunction with the intraoperative NLOI system, as described in Sec. 2.4. PBS was continuously supplied to the tissue chamber throughout the entire imaging time.

**Fig. 5:**
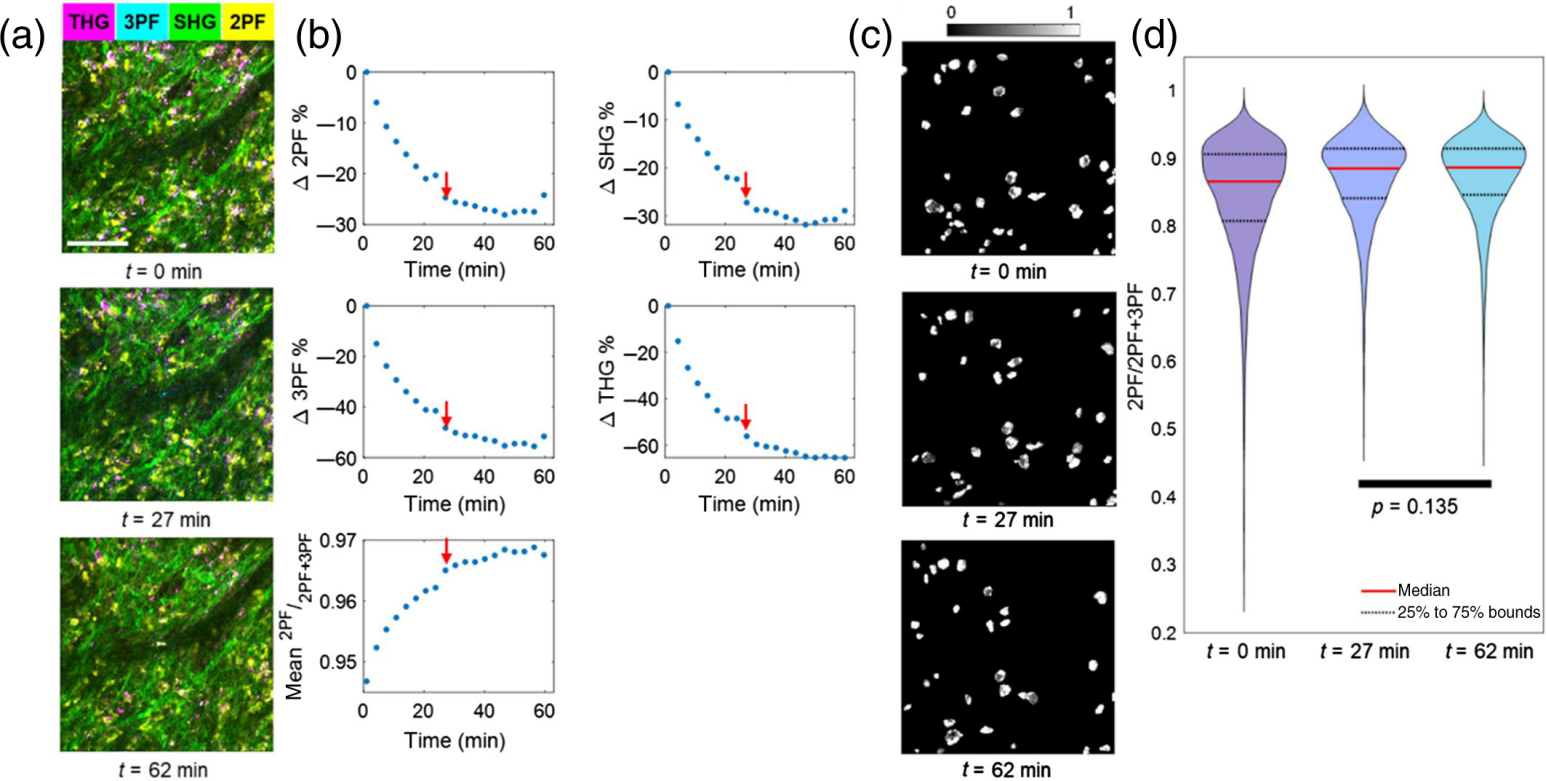
Label-free multimodal NLOI of *in vivo* biopsy taken from a rat mammary tumor and imaged in a tissue chamber with PBS perfusion. (a) Multimodal images acquired at t=0, 27, and 62 min, showed marginal structural changes. (b) Changes of ORR and channel intensities averaged over a whole frame, including the percentage changes in 2PF, 3PF, THG, and SHG mean intensities and absolute value changes of ORR. (c) ORR images after C*ellpose* segmentation at t=0, 27, and 62 min. (d) Distributions of the pixel-level ORR calculated from the segmented FOVs. Red arrows indicated t=27  min. (c) at three different time points showed changes in the metabolic profile. Kruskal–Wallis test was performed to calculate p-values. All p-values less than 0.001 were eliminated for brevity. Scale bar represents 100  μm, for all images.

Figure 5 shows the results of label-free multimodal NLOI of an NB specimen extracted *in vivo* from a rat mammary tumor, placed in the tissue culture chamber, and repeatedly imaged for 62 min. First, we investigated the changes in the tissue within the $210\times 210\;{\mu m}^{2}$ FOV. There were marginal changes in the structures captured in the same FOV [Fig. 5(a)], with an approximately 15  μm FOV shift in the perfusion flow direction (horizontal in the images). During the 62 min of imaging, the mean intensity of 2PF, 3PF, SHG, and THG decreased by 25%, 60%, 30%, and 65%, respectively, and the mean ORR increased from 0.94 to 0.97, calculated by using the entire FOV displayed in Fig. 5(a). The decreased intensity of SHG and THG could be the result of the tissue degradation, e.g., the aggregation of the collagen and the membranes, which is more visually obvious in the individually displayed images in Fig. S4 in the Supplemental Material. Though the lateral shift of the FOV was marginal, the change in the axial position was a more significant factor, attributed to tissue relaxation and flattening during its degradation. Although the axial shift was not directly measurable, it was reasonable to estimate that the shift was about 10  μm, which was about the diameter of the cells as shown in Fig. 5(a), because some of the cells were out of focus after 1-h of imaging. Additional motion artifacts were possible, as observed by the intensity discontinuity from t=24  min to t=27  min in all four channels. This could also be because the axial shift caused by tissue degradation was not smooth. Because of this continuous axial shift over time, we assume that a slightly different axial plane was illuminated for images acquired at different extended time points, and therefore helped alleviate the effect of photobleaching on the channel intensities.

The changes in the mean intensities and mean ORR can give an initial indication of the trend of tissue degradation, but there can be other contributors to the changes within the hundreds of micrometer scale. As introduced in Sec. 1, 2PF and 3PF intensities can be generated by other autofluorophores besides FAD and NAD(P)H, such as the elastin and lipids.^40^ To analyze the multiphoton autofluorescence signals from cells, an AI-based segmentation method *Cellpose*^51^ was used to automatically select the ROIs (i.e., cells) to perform the analysis of ORR. Though the algorithm can have limitations in the cases when cells are densely packed and overlapping, and when the images have poor SNR,^51^ overall, it is more reliable and less biased compared to manual segmentation. We confirmed that the trend of intensity change in each channel was not altered by the *Cellpose* segmentation (Fig. S5 in the Supplemental Material). Figure 5(c) shows the ORR images of the FOV at three time points after *Cellpose* segmentation. The distributions of the ORR were visualized in Fig. 5(d). From the start of imaging (t=0  min) to roughly the middle point (t=27  min), the average value of the ORR of the segmented FOV increased by 0.02 and IQR decreased by 0.03, and the changes were statistically significant. However, from t=27  min to the end of imaging (t=62  min), the average ORR was the same, the IQR decreased marginally by 0.005, and the changes were not statistically significant. These results indicate that during the first 27 min of imaging, the metabolic activities in the tissue within the FOV were decreasing, and then stayed almost unchanged. This suggests that this rat mammary tumor NB specimen underwent some degradation after the extraction from the intact tumor, which occurred mostly in the first 27 min.

**Fig. 6:**
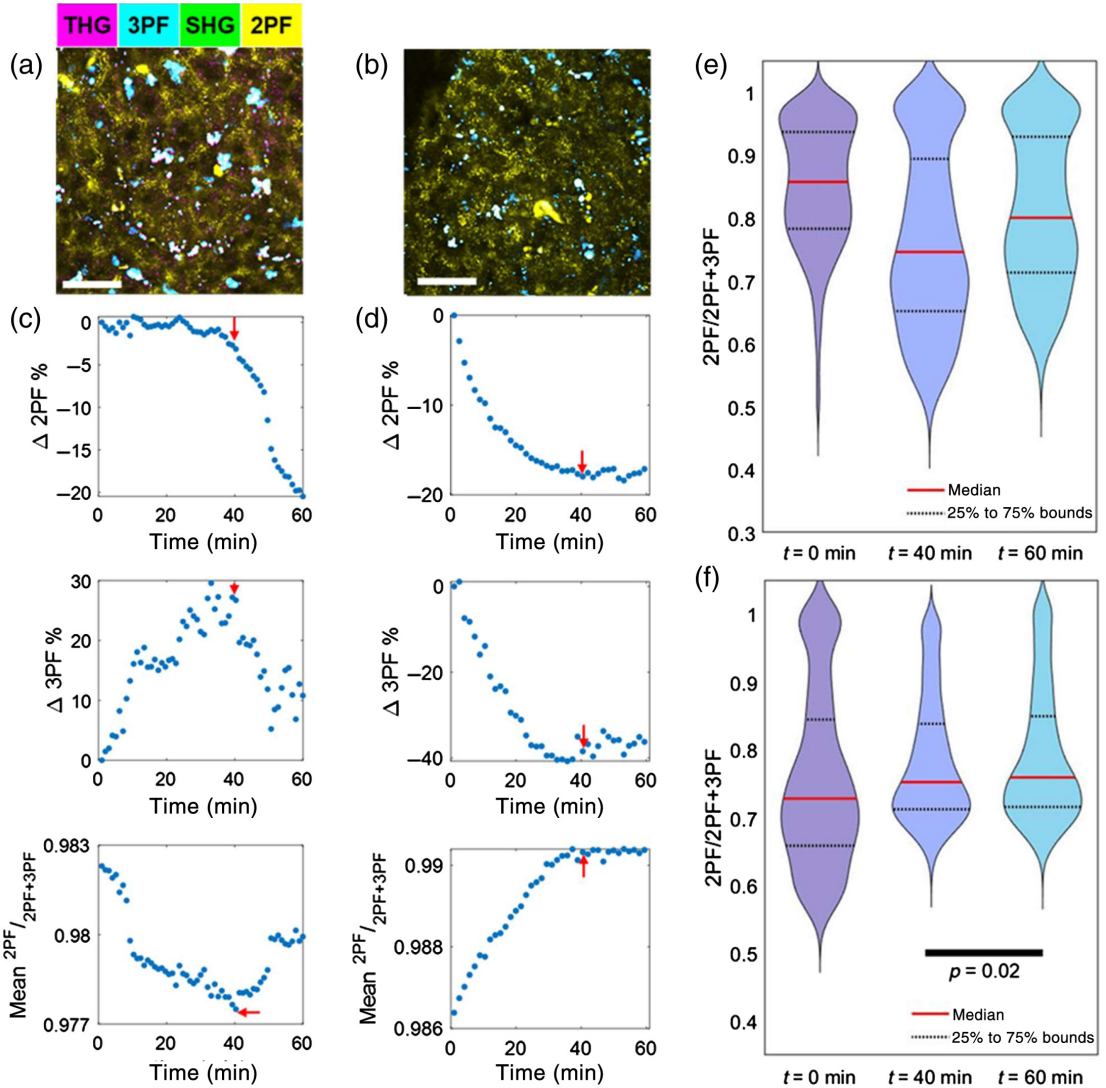
Label-free multimodal NLOI of normal mouse liver NBs specimens in a tissue chamber with PBS perfusion with (a, c, e) or without (b, d, f) the addition of 500 nM STS. (a, b) Multimodal images of a normal liver NB specimen acquired at t=0  min after the addition of STS (a) or without the addition of STS (b). (c, d) Changes of ORR and channel intensities averaged over a whole frame: (top to bottom) percentages of changes in 2PF and 3PF mean intensities and absolute value changes of the mean ORR. (c) The ORR first decreased after the addition of STS, which was mainly due to the increase of NAD(P)H. (d) The ORR increased over time, which was mainly due to faster intensity decay in 3PF. Red arrows in (c, d) indicate t=40  min. (e) Distributions of the pixel-level ORR calculated from the segmented cells in a liver NB specimen perfused with the addition of STS, at t=0, 40, and 60 min. The Kruskal–Wallis test was performed, and the p-values were all below 0.001. (f) Distributions of the pixel-level ORR calculated from the segmented FOVs in a liver NB specimen perfused only with PBS, at three different time points t=0, 40, and 60 min. The Kruskal–Wallis test was performed to calculate p-values. Only the p-value above 0.001 was displayed, all other comparisons were statistically significant (p<0.001). Scale bar represents 50  μm for all images.

After showing the degradation of NB specimens, we further investigated the tissue integrity at the start of imaging since it was not feasible to image the NB specimens right after the death of the animal. There was about 15 min time interval between the death of the animal and the start of imaging including the surgical exposure of the tumor and the extraction of the NB specimen as described in Sec. 2.5. We hypothesized that if the cells in the NB tissue microenvironment were able to respond to the apoptotic inducer added at the beginning of the imaging session, this response would indicate that the NB tissue specimens retained some degree of integrity and functionality. Under this hypothesis, to show the apoptotic responsiveness, the longitudinal imaging results of fresh murine liver NB specimens were compared with and without the addition of 500 nM STS, an apoptotic inducer, which was added to the perfusion solution PBS. Both specimens were imaged for 60 min and the multimodal images were shown in Figs. 6(a) and 6(b), respectively. The two FOVs contained similar structures, including liver sinusoids, 2PF-rich hepatocytes, lipid droplets, and some 3PF-rich cells, which were identified as hepatic stellate cells based on their morphological appearance and nonlinear signatures from other studies in the published literature.^53^^,^^54^ Figures 6(c) and 6(d) show the changes in mean 2PF and 3PF intensities and the ORR over the entire FOV. Compared to the control tissue perfused with only PBS, the addition of STS led to an increase in 3PF signal in the first 30 min, after which it decreased. This overall trend is consistent with previous observations.^36^^,^^37^ As a result, the ORR decreased with the increase of 3PF and then increased, indicating an increase in cellular metabolism with the addition of STS, followed by a decrease due to tissue degradation. In comparison, when the liver tissue was perfused only with PBS, the ORR in the FOV increased gradually in the first 40 min and did not change significantly until the end of the imaging session (60 min), which is similar to the imaging results from the rat mammary tumor NB specimen, as discussed previously.

Metabolic profiling of the liver NB specimens was performed on the images at three time points during the imaging session, after cell auto-segmentation by *Cellpose*. Figure 6(e) shows the metabolic profiles of a liver NB specimen perfused with the addition of STS, at t=0, 40, and 60 min. The middle time point was chosen based on the observation from the ORR changes within the whole frame before segmentation [Fig. 6(b), bottom graph]. The median value of the ORR in the segmented FOV decreased from 0.86 to 0.74 and then increased to 0.8, following a similar trend as shown in Fig. 6(c). The IQR increased from 0.15 to 0.24 and then decreased to 0.21. All changes were determined to be statistically significant. Comparatively, metabolic profiles of the liver NB specimens perfused with PBS only, as shown in Fig. 6(f), display a similar trend as in Fig. 5(d). There was a statistically significant increase in the median value of the ORR from 0.72 to 0.75, and a decrease in IQR from 0.19 to 0.13 in the first 40 min, which indicates slowing of cellular metabolism. However, from t=40 to 60 min, changes both in median ORR and the IQR were <0.01, and the statistical significance was much lower. The comparison between the two perfusion conditions implies that the freshly extracted liver NB specimens had a certain degree of functionality, since there was a response to the apoptosis inducer, and without the intervention of STS, the fresh NB specimens slowly degraded over time. This comparative experiment further confirms the significance of using a label-free imaging tool for the intraoperative assessment of NB specimens at the point-of-procedure. In addition, the combination of an intraoperative label-free multimodal NLOI system with a tissue chamber and perfusion system could potentially be used for studying more immediate real-time responses to various chemotherapies or interventional drugs on the fresh NB specimens.

### 3.3 Limitations and Future Directions

We have demonstrated the ability of an intraoperative label-free multimodal NLOI system to provide histology-like morphology information about the tissue microenvironment, as well as quantitative characterizations of the microstructures, metabolic activities, tissue degradation, and response to an applied drug agent. However, the limitations of the current system are worth noting. Details of the system hardware limitations can be found in the previously published work,^46^ among which the excitation beam characteristics (i.e., pulse-width and repetition rate) account for the bottleneck of the system performance in terms of SNR and speed. Replacement of the currently used commercial laser source with one that has a lower repetition rate, and the addition of an external pulse compression module, can improve the SNR and thus the imaging speed. However, the current system was built to be a compact, relatively less costly, turn-key cart system to enable intraoperative imaging. It was also experimentally estimated that the image SNR needed for reliable ORR analyses was around 40 photons for fluorescence intensity.^54^^,^^55^ In the data presented here, the accuracy of the ORR analysis is mainly limited by the 3PF signals. The three-photon absorption cross-section is known to be orders of magnitude smaller than the two-photon absorption cross-section,^56^ which intrinsically limits 3PF signal strengths. In the data presented in this work, the ORR was generally close to 1 because of the weaker 3PF signals, which resulted in small absolute value changes in ORR.

The accuracy of the metabolic profiling method described above is not only limited by the SNR but also by the specificity. It is known that lipids and vitamin A have overlapping autofluorescence emission spectra with that of NAD(P)H. With only the fluorescence intensity signals, it is difficult to exclude the contributions of both lipids and vitamin A from the 3PF autofluorescence of NAD(P)H, which can affect the accuracy of the ORR. The cell differentiation method used in this work was based on morphology, e.g., size and shape. The addition of more specific imaging modalities such as FLIM^57^ and nonlinear Raman imaging^58^ can be used to better distinguish cellular NAD(P)H from other spectrally overlapping endogenous fluorophores, and thus give a more accurate metabolic profile of the tissue microenvironment. The use of the ORR for measuring relative metabolic changes was not affected by the emission spectrum overlap of FAD and NAD(P)H^59^ because the 3PF of NAD(P)H, in general, was much weaker in intensity, and the 3PF spectral filter window cuts off at 470 nm, which should limit the FAD fluorescence leakage into the 3PF channel.

Besides limitations in the imaging system performance, we also recognized the complications in tissue degradation. For example, we were not able to quantify the changes in the mechanical properties of the tissue undergoing degradation, e.g., the relaxation and flattening. Although there are optical elastography techniques such as optical coherence elastography^60^ and Brillouin microscopy^61^ which have been used for experimentally measuring the mechanical properties in unstained tissue, these techniques are challenging, practically, to be integrated into a multimodal NLOI system. However, auto-focusing can be implemented to counter the axial shift due to tissue degradation. Image registration during post-processing of three-dimensional datasets can also be performed to alleviate this problem. Moreover, the baseline perfusion solution used in this work was PBS. This solution was chosen because it is most commonly used in the lab during the transportation of tissue specimens from the point of extraction to the imaging stage. However, other solutions such as tissue-specific culture media (details listed in Fig. S1 in the Supplemental Material) can potentially sustain the tissue viability and integrity better and will be investigated in the future with this NLOI system. Although only one type of apoptotic inducer was used to representatively demonstrate the ability of the intraoperative NLOI system to detect drug responsiveness of NB specimens, various other chemotherapeutic drugs can be applied in the future for testing drug responses of fresh NB specimens at the point-of-procedure. Eventually, intraoperative NLOI of the human NB specimens will be performed using the improved system.

## 4 Conclusion

In this paper, we demonstrated the use of an intraoperative label-free multimodal NLOI system for imaging fresh canine NB tissue cores acquired during surgical procedures to remove the tumors. Furthermore, with the addition of a tissue chamber, this portable NLOI system has the potential to monitor the degradation of tissue integrity and even study drug response over time through longitudinal imaging, which was shown in this study for fresh murine NB cores from healthy and induced-cancer animal models. Despite the system limitations on speed and SNR as a compromise for system compactness and turn-key operation, this portable imaging system has the capability to provide quantitative microstructural and metabolic information of the unperturbed tissue environment in fresh NB tissue cores, as well as dynamic information about the tissues and cells. We believe that with future enhancement in system SNR and speed, this system and this multimodal label-free imaging approach can potentially be used as a diagnostic assessment tool for NB specimens at the point-of-procedure and provide a more comprehensive understanding of the unperturbed tumor microenvironment for both human and companion animals.

## Code, Data, and Materials Availability

The code and data presented in this manuscript can be made available from the corresponding author through a collaborative research project.
