# Supplementary material for: Label-free multimodal nonlinear optical imaging of needle biopsy cores for intraoperative cancer diagnosis
Source: J Biomed Opt. 2022 May 28;27(5):056504. doi: 10.1117/1.JBO.27.5.056504 (PMC9142840; doi:10.1117/1.JBO.27.5.056504)
Supplement: Supplementary file 1 [file JBO_027_056504_SD001.pdf]

Label-Free Multimodal Nonlinear Optical Imaging of Needle Biopsy Cores for Intraoperative Cancer Diagnosis

Lingxiao Yang,[a,b] Jaena Park,[b,c] Eric J. Chaney,[b] Janet E. Sorrells,[b,c]
Marina Marjanovic, [b,c,e] Heidi Phillips,[d] Darold R. Spillman Jr.,[b]
Stephen A. Boppart[a,b,c,e,f,*]

[a] University of Illinois at Urbana-Champaign, Department of Electrical and Computer Engineering, Urbana, IL, USA
[b] University of Illinois at Urbana-Champaign, Beckman Institute for Advanced Science and Technology, Urbana, IL, USA
[c] University of Illinois at Urbana-Champaign, Department of Bioengineering, Urbana, IL, USA
[d] University of Illinois at Urbana-Champaign, College of Veterinary Medicine, Urbana, IL, USA
[e] University of Illinois at Urbana-Champaign, Carle Illinois College of Medicine, Champaign, IL, USA
[f] University of Illinois at Urbana-Champaign, Cancer Center at Illinois, Urbana, IL USA

Supplemental Material

Table S1. Demographic information about the canine patients enrolled in the study.

Type of tumor	Number of cases	Breed	Age (years)
Liver	4	Poodle	14
		Yorkshire terrier	9
		Mixed breed	11
		Mixed breed	11
Sarcoma	2	Boxer	7
		Labrador retriever	12
Lung	1	Golden retriever	10
Mammary	1	Mixed breed	10

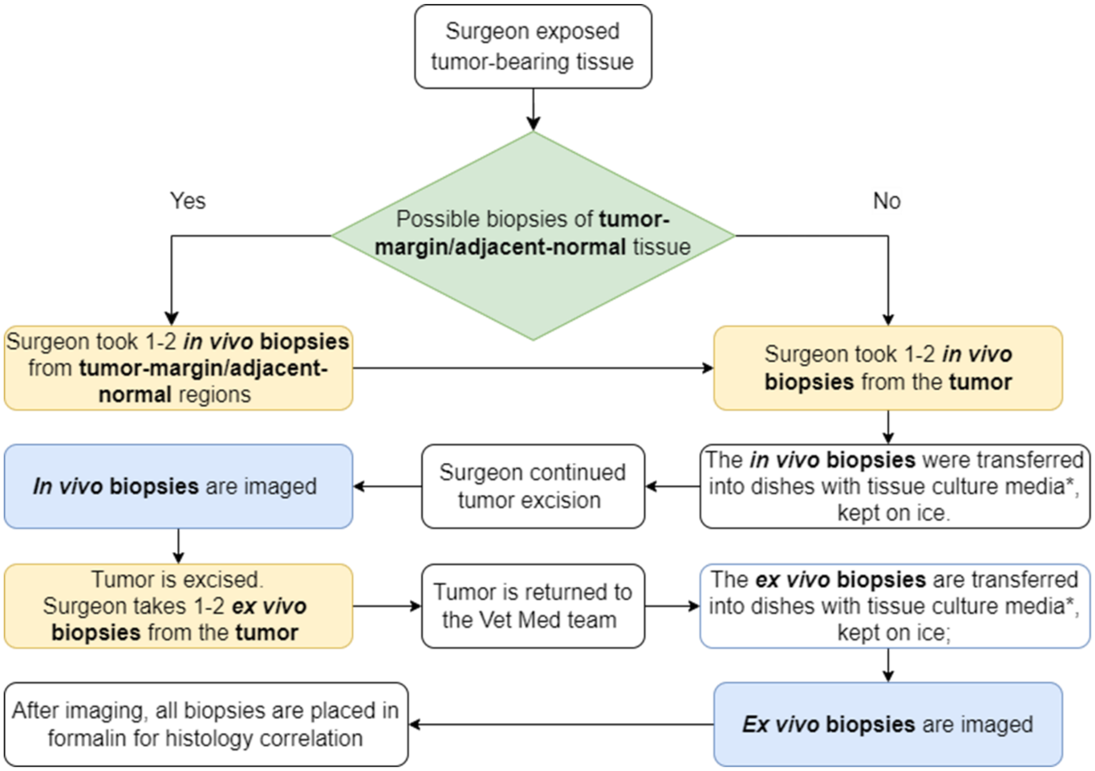

Tissue type	Tissue culture media
**Liver**	William's E medium with primary hepatocyte maintenance supplements, GlutaMAX, and HEPES
**Lung**	RPMI medium, without phenol red
**Mammary**	MEM-alpha with 25 mM HEPES, without phenol red
**Soft tissue**	DMEM with 25 mM HEPES, without phenol red, supplemented with 10% FBS and 1% penicillin-streptomycin

Fig. S1. Flow chart of the intraoperative NLOI procedure during canine cancer surgeries. In this chart, *in vivo* biopsies refer to NB specimens taken from an intact organ or tumor of a live animal during surgery, and before surgical removal of that organ or tumor. In contrast, *ex vivo* biopsies refer to the NB specimens taken from the excised organ or tumor. The table lists the composition of the tissue culture media for different types of tissue, namely liver, lung, mammary and soft tissues.

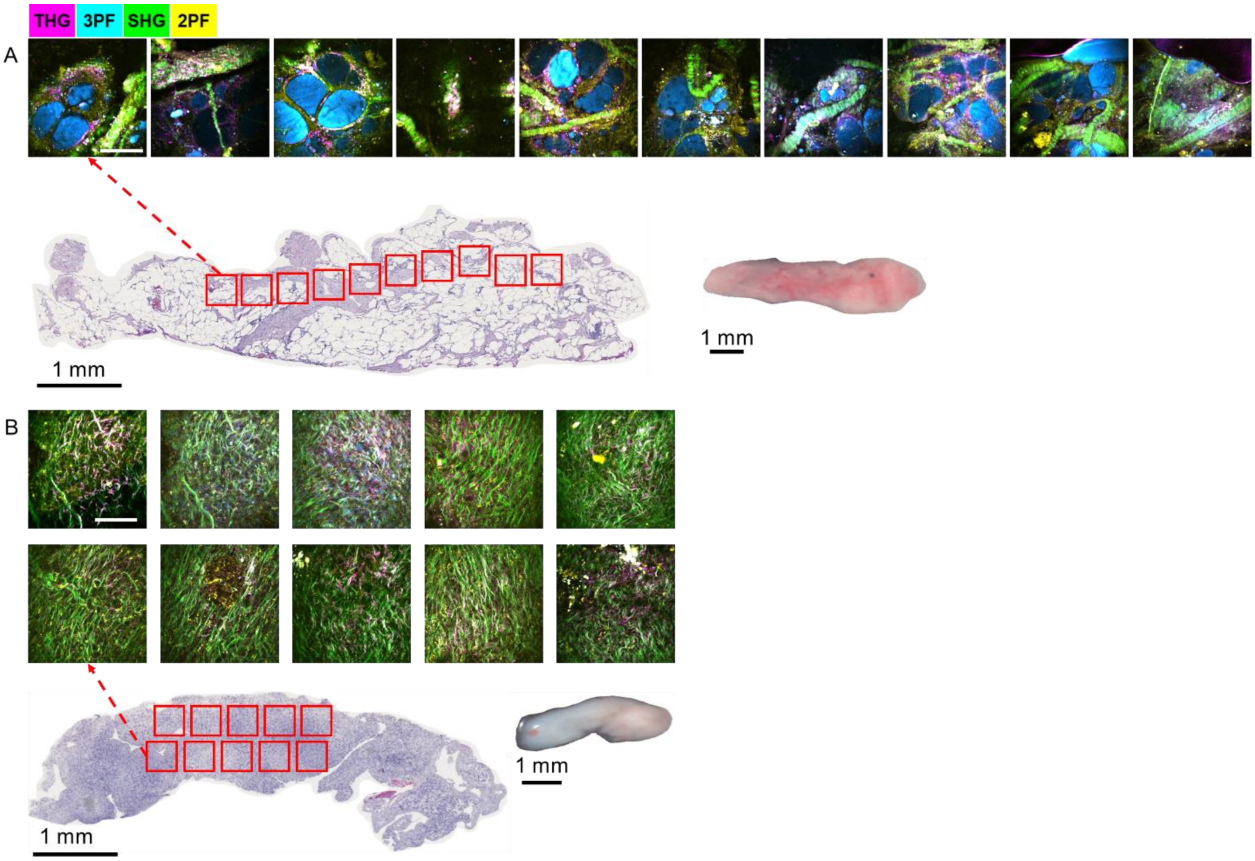

Fig. S2. Intraoperative label-free multimodal NLOI of canine NB tissue cores taken *in vivo* from the (A) tumor margin and the (B) tumor region in a soft tissue sarcoma. The camera images (bottom right) were used to guide the ROI selection. In the case of a tumor margin (A), the ROIs were selected along the vessel-like red region based on the camera image. For the tumor NB core (B), since the camera image appeared to be homogeneous, the ROIs were chosen to cover the central region of the NB core. The NLOI ROIs were mapped to the histology images (bottom left). Scale bars for NLOI represent 100 µm.

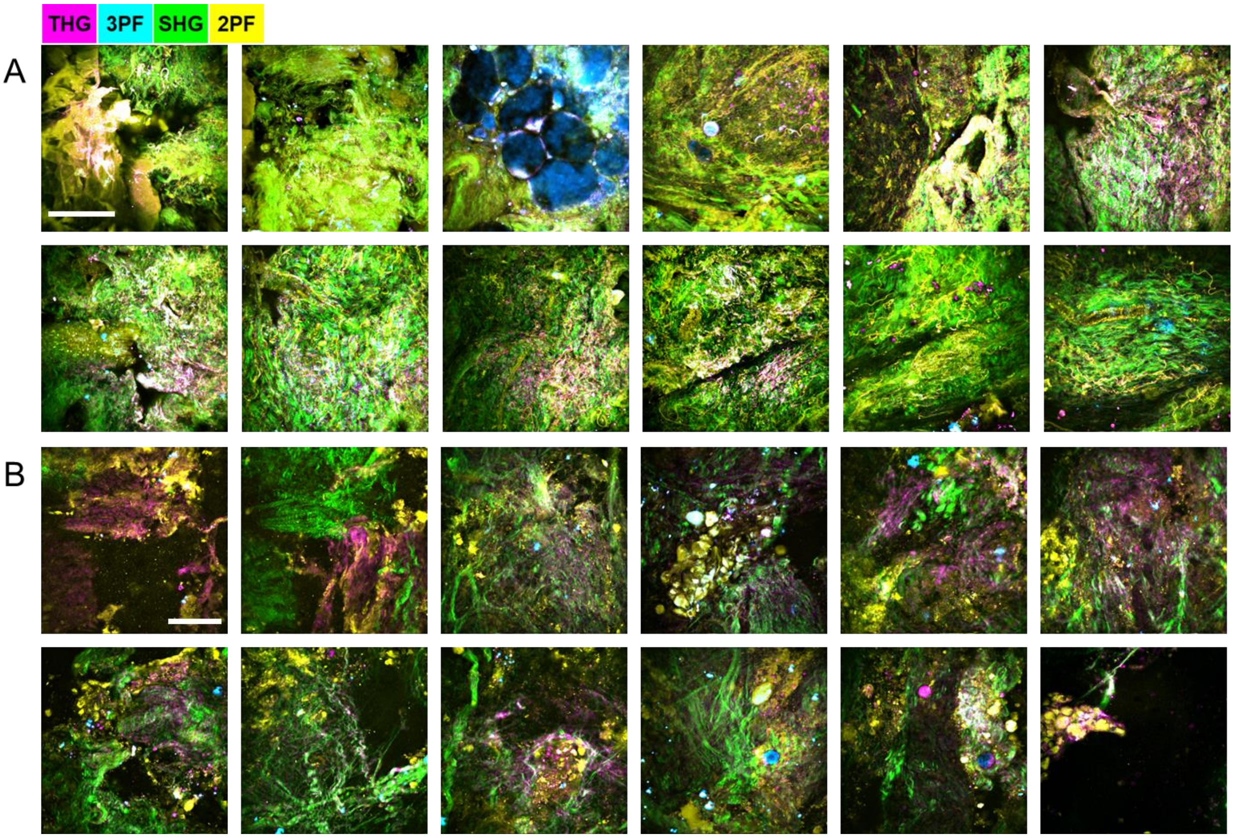

Fig. S3. Intraoperative label-free multimodal NLOI of a mammary NB tissue core extracted *in vivo* from a canine patient. (A) Enlarged images from a tumor margin NB core as shown in Fig. 2, and images from a mammary tumor NB core. The quantitative SHG analysis was performed on this dataset and the comparison with the tumor margin was shown in Fig. 3. Scale bar represents 100 μm.

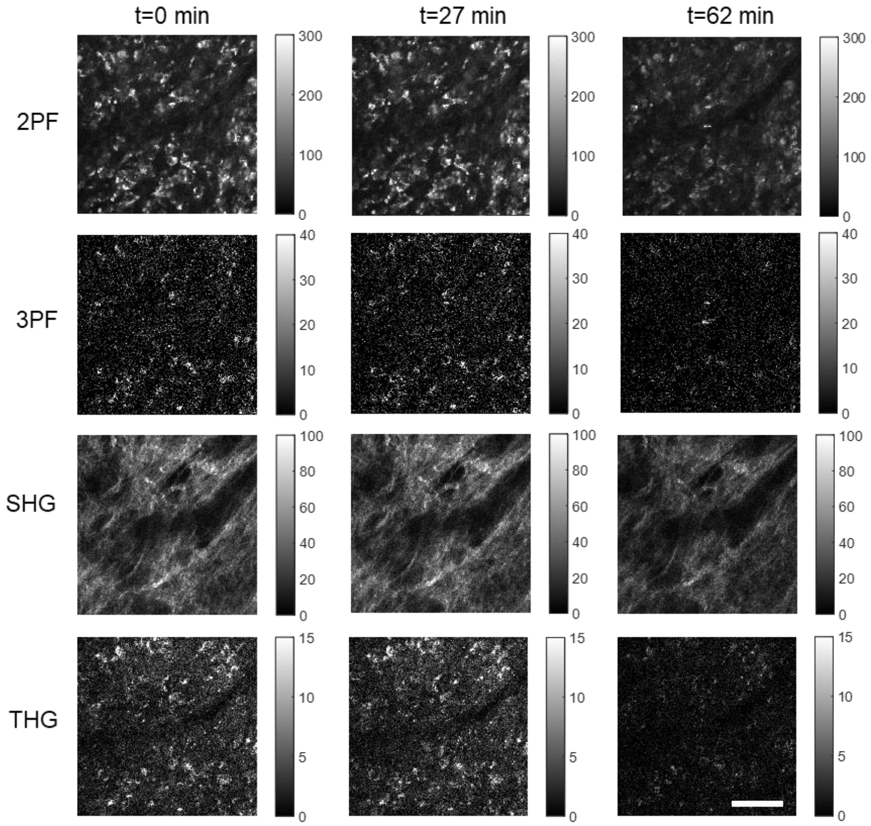

Fig. S4. Individual channel images of the multimodal NLOI results in Fig. 5(A) were acquired at three timepoints (t= 0 min, t=27 min, and t=62 min) and displayed using the same scale across different timepoints for better visualization of the intensity decreases in all channels. Scale bar represents 100 µm.

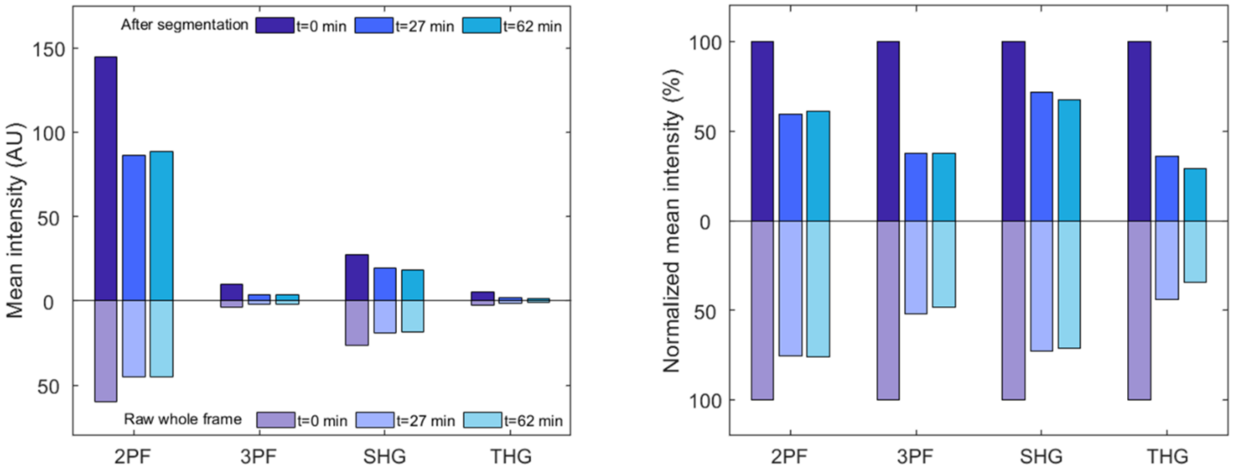

Fig. S5. Comparison of the mean value changes of the image intensities in all four channels from t=0 min, to t=27 min and t= 62 min before and after the *Cellpose* segmentation.

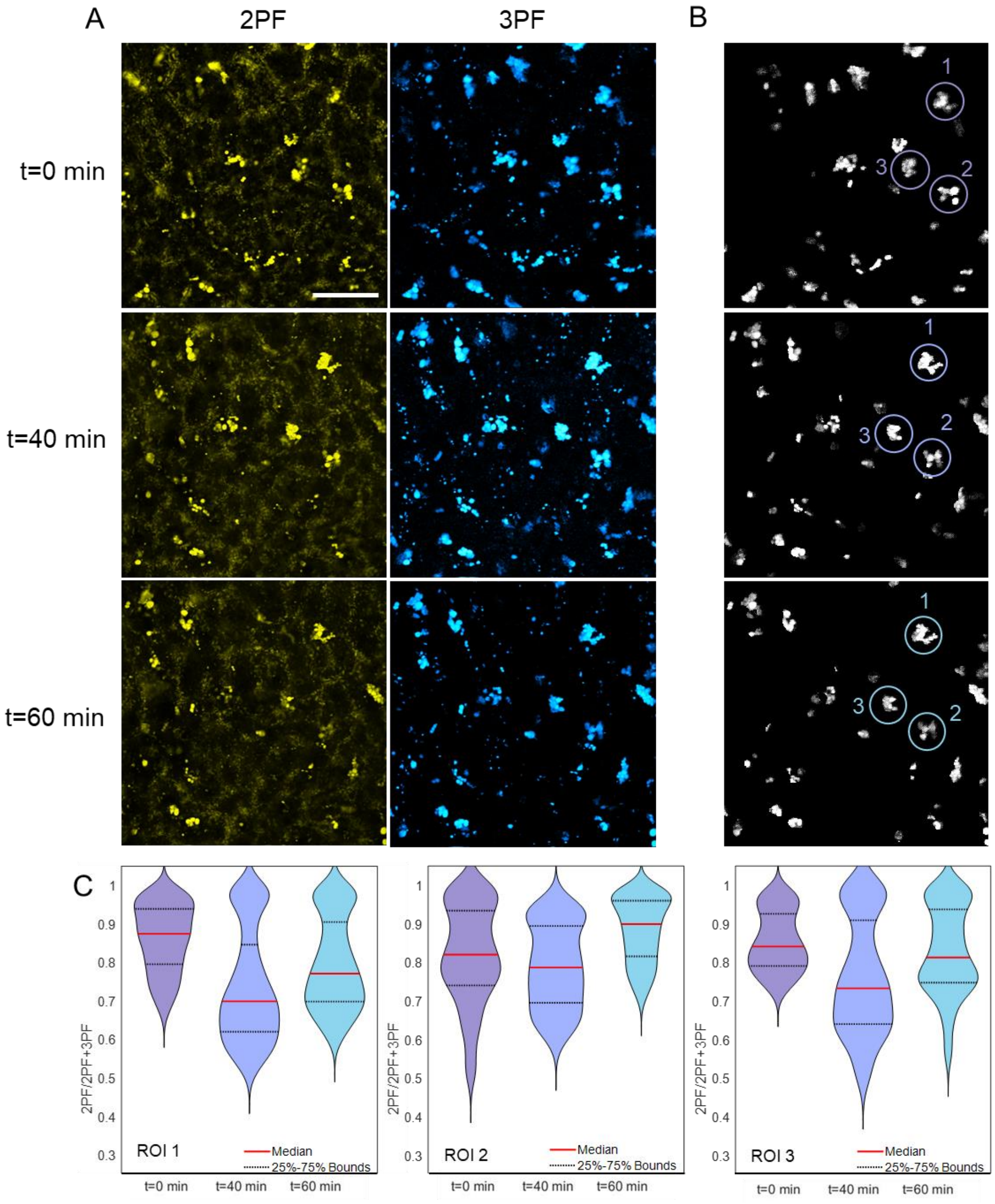

Fig. S6. Longitudinal NLOI of a fresh liver NB core sustained in a tissue chamber perfused with PBS and 500 nM STS. (A) 2PF and 3PF intensity images of the approximately same FOV at t=0 min, 40 min, and 60 min. (B) Optical redox ratio image after *Cellpose* segmentation at t=0 min, 40 min, and 60 min. Three smaller ROIs (circles) were selected for ORR distribution analysis shown in (C). (C) Redox ratio distribution of selected ROIs changed from t=0 min, 36 min to 52 min. The Kruskal-Wallis test was performed to calculate p-values between each group and all p-values were less than 0.001. Bars indicating statistical significance between groups were omitted from the figure for brevity. Scale bar represents 50 μm.

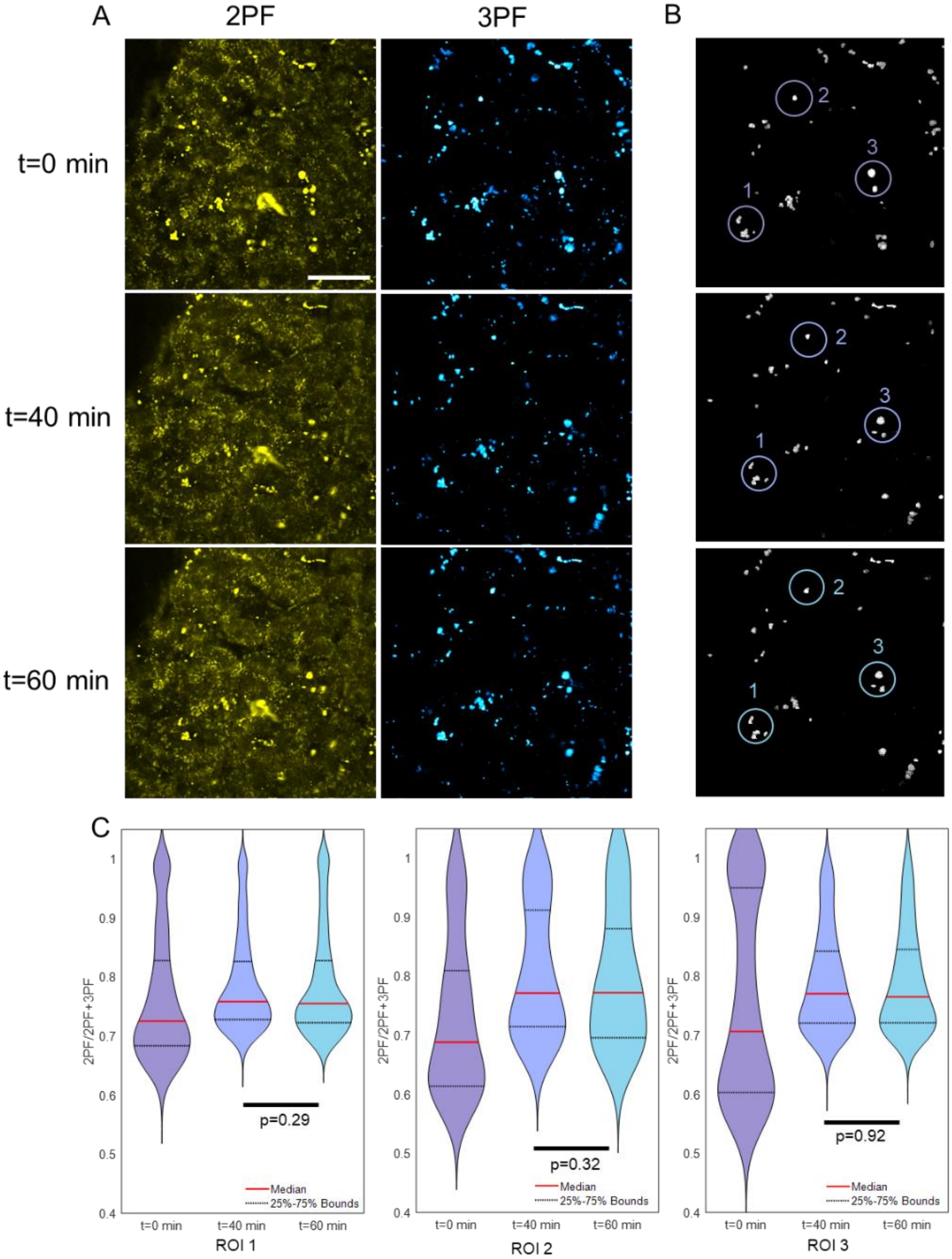

Fig. S7. Longitudinal NLOI of a fresh liver NB core sustained in a chamber perfused with only PBS. (A) 2PF and 3PF intensity images of the approximately same FOV at t=0 min, 40 min, and 60 min. (B) Optical redox ratio image after *Cellpose* segmentation at t=0 min, 40 min, and 60 min. Three smaller ROIs (circles) were selected for ORR distribution analysis shown in (C). (C) Redox ratio distribution changed from t=0 min, 40 min to 60 min. The

Kruskal-Wallis test was performed to calculate p-values between each group and the p-values less than 0.001 were omitted from the figure for brevity. In all three ROIs, the changes from t = 0 to 40 min were statistically significant but the changes from t = 40 min to 60 min were not. Scale bar represents 50 μm.
